# One-Hour Bundle Protocols for Surgical Sepsis and Septic Shock in Surgical Intensive Care Unit: Clinical Outcome Aspects in the Thai Context

**DOI:** 10.7759/cureus.62215

**Published:** 2024-06-12

**Authors:** Panu Boontoterm, Siraruj Sakoolnamarka, Karanarak Urasyanandana, Pusit Fuengfoo

**Affiliations:** 1 Neurological Surgery, Phramongkutklao Hospital, Bangkok, THA; 2 Surgery, Phramongkutklao Hospital, Bangkok, THA

**Keywords:** surviving sepsis campaign, perioperative period, median survival time, surgical sepsis, one-hour bundle care

## Abstract

Background: Surgical sepsis is a syndrome occurring during the perioperative period with a high mortality rate. Since the one-hour bundle protocol was recommended to decrease sepsis-related morbidity and mortality in clinical practice, the protocol has been applied to surgical patients with sepsis and septic shock. However, clinical outcomes in these surgical patients remain unknown. Thus, this study aimed to compare survival outcomes in patients before and after the implementation of one-hour bundle care in clinical practice.

Methods: In this prospective cohort study, 401 surgical patients with sepsis were divided into two groups, with 195 patients undergoing the one-hour bundle from December 25, 2021, to March 31, 2024, and 206 patients undergoing usual care from January 1, 2018, to December 24, 2021, before the one-hour bundle protocol was implemented by the Surviving Sepsis Campaign (SSC). Demographic data, treatment processes, and clinical outcomes were recorded.

Results: After the one-hour bundle protocol was applied in surgical practice, the median survival time was significantly increased in surgical patients who underwent one-hour bundle care (95% confidence interval (CI): 12.32-19.68) (p= 0.016). Factors influencing the increase in the mortality rate were delays in fluid resuscitation of >2 hours, vasopressor initiation of >2 hours, and empirical antibiotics of >5 hours (p= 0.017, 0.028, and 0.008, respectively).

Conclusion: One-hour bundle care for surgical patients with sepsis resulted in an increased median survival time. Delays in fluid resuscitation (>2 hours), vasopressor initiation (>2 hours), and empirical antibiotics (>5 hours) were factors associated with mortality.

## Introduction

Sepsis is a common medical problem in the intensive care unit (ICU). In surgical ICU patients, sepsis from surgical site infection causes a high morbidity and mortality rate. A recent study from the American College of Surgeons found that both sepsis and septic shock are 10 times more common than intra- and postoperative myocardial infarction and pulmonary embolism [1-3]. In addition, sepsis-related deaths occur each year, despite advancements in the management of surgical critical care [4,5]. Sepsis in surgical patients during the perioperative period is still a common and impactful problem in patient care, and its incidence continues to increase each year. Early identification of sepsis, especially in surgical patients, is crucial.

The impact of a sepsis screening practice guideline, together with a one-hour bundle protocol for the application of sepsis-related medical management and early source control in surgical patients, has improved patient survival. The Surviving Sepsis Campaign (SSC) launched the one-hour bundle as an initiative for the management of both sepsis and septic shock to reduce mortality rates; it is additionally recommended by several societies in intensive care medicine [6,7]. The SSC one-hour bundle includes the most recent advancements in patient care and early therapeutic treatment to be imposed as a one-hour fluid resuscitation, including blood lactate examination, blood cultures, empirical antibiotics, and a 24-hour care map for sepsis and septic shock, which encompasses controlling glycemic levels, optimizing plateau pressure limitation, and considering intravenous hydrocortisone treatment [8-12].

In a surgical ICU setting, intra-abdominal infection is the most common cause of sepsis and septic shock, accounting for about two-thirds of cases among all surgical patients [13]. Large-intestine perforation, especially that in the colon, is the most common source of intra-abdominal sepsis [14]. When sepsis progresses to septic shock in surgical patients, a 39% mortality rate is reported among emergency surgical procedures, and a 30% mortality rate is reported among elective surgical procedures. The one-hour bundle demonstrated that improvement in the survival of patients with sepsis is time-sensitive. Administration of empirical broad-spectrum antibiotics is strongly recommended within one hour of clinical suspicion, as each hour of delay in the administration of empirical antibiotic therapy is associated with an increase in mortality rate. However, early management requires early detection of sepsis. A recent study [15] demonstrated a significant correlation between time and proper empirical antibiotic administration and patient survival outcomes [16]. Among patients having major surgical oncologic procedures, 20.4% were diagnosed with sepsis, and the mortality rate of these patients was 36.8% [17,18]. In patients who had intra-abdominal infections and underwent surgery to explore abdominal laparotomy, 11% had sepsis. Multiple-organ dysfunction was most commonly found in the respiratory, kidney, and cardiovascular systems. The mortality rate was 34% in surgical patients diagnosed with sepsis, compared with 6% overall in the general population [19].

This study aimed to compare the mortality rates, median survival times, and treatment outcomes of patients before and after the implementation of the one-hour bundle.

## Materials and methods

In this prospective cohort study, 401 patients were divided into two groups, with 195 undergoing the one-hour bundle from December 25, 2021, to March 31, 2024, and 206 patients undergoing usual care from January 1, 2018, to December 24, 2021, before the one-hour bundle was implemented by the SSC. Data collection was composed of patient demographic data, treatment processes, and clinical outcomes, using data from a recent prospective study comparing sepsis and septic shock mortality before and after the one-hour bundle was implemented in the surgical ICU at Phramongkutklao Hospital. The pre-intervention cohort included all consecutive surgical patients with sepsis and septic shock admitted to the surgical ICU.

The one-hour bundle

The SSC protocol recommends early management of sepsis and septic shock as a "one-hour bundle." Fluid resuscitation, which is initiated immediately and started within the first hour of sepsis and septic shock presentation, included obtaining serum lactate levels, performing hemoculture prior to empirical intravenous antibiotic administration, and administering intravenous broad-spectrum antibiotics within one hour of clinical suspicion of sepsis at emergency department admissions. "Post-resuscitation management," which is completed within the first 24 hours of the presentation of clinical clues of sepsis and septic shock, included considering the administration of intravenous hydrocortisone (200 mg), maintaining glycemic control at a median range of 80-180 mg/dL, and maintaining an inspiratory plateau pressure of ≤30 cm H2O in surgical patients with sepsis or septic shock who require mechanical ventilators.

In both the pre- and post-one-hour bundle implementation in this study, all surgical ICU admissions from the emergency department or inpatient department and all surgical ICU patients were actively screened daily for the presence of sepsis or sepsis progression to septic shock. Patients were systematically defined using a screening tool that included definitions of sepsis and multiple-organ dysfunction (Sequential Organ Failure Assessment Scores (SOFA) and Sepsis-3, defined as a persistent mean arterial pressure (MAP) of <65 mmHg for ³15 minutes, despite adequate volume resuscitation (dynamic parameters showing fluid non-responsiveness)) or based on the requirement of a vasopressor to maintain a MAP of >65 mmHg and serum lactate level of >2 mmol/L. Patients for whom the onset of severe sepsis (time 0) could not be determined were not included in the study.

Data collection

The clinical outcomes and baseline characteristics of all patients, including age, sex, Acute Physiology and Chronic Health Evaluation II (APACHE II) score, SOFA score, and source of infection, were collected. Hospital and ICU lengths of stay were recorded. Moreover, compliance with the different elements of the one-hour bundle protocol was recorded.

Statistical analysis

Descriptive statistics included frequencies and percentages for categorical variables and parameters, as well as means and standard deviations (SDs), both lower and upper, for continuous variables. To compare variables during the two study periods, the independent t-test or chi-square tests were used when appropriate. The primary outcome was median survival time before and after the implementation of the one-hour bundle, and the secondary outcomes were differences in ICU length of stay, ventilator use, vasopressor-free days, and factors associated with mortality. In a previous pilot study by Brun-Buisson et al. [2] investigating the incidence, risk factors, and outcomes of sepsis and septic shock in adults, sample size estimation showed that >225 patients were required to evaluate and compare treatment protocol and usual care. Results were demonstrated as means ± SDs if data and variables were normally distributed or medians and interquartile ranges (IQRs) if they were not normally distributed. A p-value of <0.05 was considered to be statistically significant. Statistical analysis was performed using SPSS version 25.0 (IBM SPSS Statistics, Armonk, NY).

Ethics approval and consent to participate

The Thai Clinical Trials Registry Committee approved the study under their opinion number TCTR20240509003 on May 9, 2024, and the Ethics Committee of the Institutional Review Board of the Royal Thai Army Medical Department approved this study on December 24, 2021. Research number S068h/64 followed the Council for International Organization of Medical Science (CIOMS) Guidelines 2012 and Good Clinical Practice of International Conference on Harmonization statement number IRBRTA 1861/2564. Because this was a prospective study with no specific intervention and only the use of deidentified medical records, inpatient department data, and other hospital clinical data, written informed consent was obtained according to the CIOMS Guidelines 2012 and Good Clinical Practice of International Conference on Harmonization.

## Results

Patient characteristics

A total of 401 patients fulfilled the sepsis or septic shock criteria for diagnosis owing to the Sepsis-3 definition and were therefore included in the study. The one-hour bundle group included 195 patients with the following characteristics: mean (SD) age, 65.17 (16.22) years; mean (SD) APACHE II score, 13.55 (1.5); mean (SD) SOFA score, 6.75 (1.01); male sex, 65.1% (n = 127); origin in gastrointestinal tract, 55.4% (n = 108); and hospital mortality, 14.27 ± 6.89 days. The usual care group included 206 patients with the following characteristics: mean (SD) age, 63.48 (16.29) years; mean (SD) APACHE II score, 13.63 (1.56); mean (SD) SOFA score, 6.8 (1.06); male sex, 64.6% (n = 133); origin in gastrointestinal tract, 54.4% (n = 108); and hospital mortality, 6.43 ± 6.63 days. The demographic data of patients in both groups were comparable (Table 1), with no statistically significant differences in age, sex, body mass index, underlying disease (hypertension, dyslipidemia, diabetes mellitus, chronic kidney disease, coronary artery disease, malignancy, and cerebrovascular accident), APACHE II score, SOFA score, serum lactate and albumin at admission, number of organ systems involved, specific organ sepsis, or number of organ failures. The most frequent coexisting disease was hypertension, and the most frequent specific organ sepsis was that of the gastrointestinal tract.

**Table 1:** Baseline characteristics between the two patient groups Values are presented as mean ± SD or number (%). p-value corresponds to the independent t-test and chi-square test. BMI: body mass index, SOFA: Sequential Organ Failure Assessment, APACHE II: Acute Physiology and Chronic Health Evaluation II, SSI: surgical site infection, SD: standard deviation

	One-hour bundle (n = 195)	Usual care (n = 206)	p-value
Sex			
Male	127 (65.1%)	133 (64.6%)	0.906
Age (year)	65.17 ± 16.22	63.48 ± 16.29	0.298
BMI (kg/m^2^)	22.41 ± 2.83	22.24 ± 2.71	0.547
Hypertension	139 (71.3%)	144 (69.9%)	0.762
Dyslipidemia	70 (35.9%)	72 (35%)	0.843
Diabetes mellitus	111 (56.9%)	119 (57.8%)	0.864
Chronic kidney disease	12 (6.2%)	12 (5.8%)	0.890
Coronary artery disease	60 (30.8%)	63 (30.6%)	0.968
Malignancy	71 (36.4%)	73 (35.4%)	0.839
Cerebrovascular accident	10 (5.1%)	10 (4.9%)	0.900
SOFA score	6.75 ± 1.01	6.8 ± 1.06	0.649
APACHE II score	13.55 ± 1.5	13.63 ± 1.56	0.590
Serum lactate at admission	5.39 ± 1.26	5.42 ± 1.37	0.878
Serum albumin at admission	2.67 ± 0.49	2.69 ± 0.49	0.689
Number of organ systems involved			
1	155 (79.5%)	158 (76.7%)	0.500
2	40 (20.5%)	48 (23.3%)	
Specific organ sepsis			
Gastrointestinal tract	108 (55.4%)	112 (54.4%)	0.997
SSI	67 (34.4%)	72 (35%)	
Urinary tract	10 (5.1%)	11 (5.3%)	
Cranial and spine	10 (5.1%)	11 (5.3%)	
Number of organ failure	1.37 ± 0.57	1.38 ± 0.55	0.800

Adherence to both the resuscitation and one-hour bundle protocol was greater in the treatment group than that in the usual care group owing to SSC guidelines (Table 2). Moreover, in the usual care group, the mean time to initial fluid resuscitation, vasopressor use, performance of blood culture and sensitivity, initiation of empirical antibiotics, and source control were 2.33 ± 0.58 hours, 3.52 ± 0.84 hours, 4.17 ± 0.5 hours, 5.16 ± 0.54 hours, and 10.32 ± 0.66 hours, respectively. Table 2 presents, among other results, that adherence to both the resuscitation and one-hour bundle correlated with a shorter hospital length of stay, lower cost of treatment, shorter ICU length of stay, and higher mortality days in the treatment group than those in the usual care group owing to SSC guidelines. Additionally, adherence to both the resuscitation and one-hour bundle correlated with higher ventilator- and vasopressor-free days in the treatment group than those in the usual care group. The overall mean cost per patient was 48,005.35 Thai Baht in the treatment group and 62,919.37 Thai Baht in the usual care group, suggesting that an increase of >14,914 Thai Baht per patient was mainly attributed to the increased hospital length of stay. The risk of hospital mortality was lower in the one-hour bundle group than that in the usual care group (6.43 ± 6.63 versus 39.7%) (p < 0.001).

**Table 2:** Difference of secondary outcomes between the two patient groups Values are presented as mean ± SD or number (%). p-value corresponds to the independent t-test and chi-square test. ICU: intensive care unit, SD: standard deviation

	One-hour bundle (n = 195)	Usual care (n = 206)	p-value
Initial fluid resuscitation (hours)	1 ± 0	2.33 ± 0.58	<0.001*
Vasopressor (hours)	1 ± 0	3.52 ± 0.84	<0.001*
Culture (hours)	1 ± 0	4.17 ± 0.5	<0.001*
Initiated empirical antibiotics (hours)	1 ± 0	5.16 ± 0.54	<0.001*
Source control (hours)	4 ± 1.41	10.32 ± 0.66	<0.001*
Hospital length of stay (days)	7.91 ± 1.68	16.8 ± 3.77	<0.001*
Cost of treatment (Thai Baht)	48,005.35 ± 9,598.83	62,919.37 ± 1,186.29	<0.001*
ICU length of stay (days)	3.88 ± 1.37	11.07 ± 4.38	<0.001*
Ventilator-free days (days)	5.59 ± 1.9	1.67 ± 0.82	<0.001*
Mortality days (days)	14.27 ± 6.89	6.43 ± 6.63	<0.001*
Vasopressor-free days (days)	7.25 ± 1.81	3 ± 0	<0.001*

Differences in secondary outcomes between the two groups

**Figure 1:**
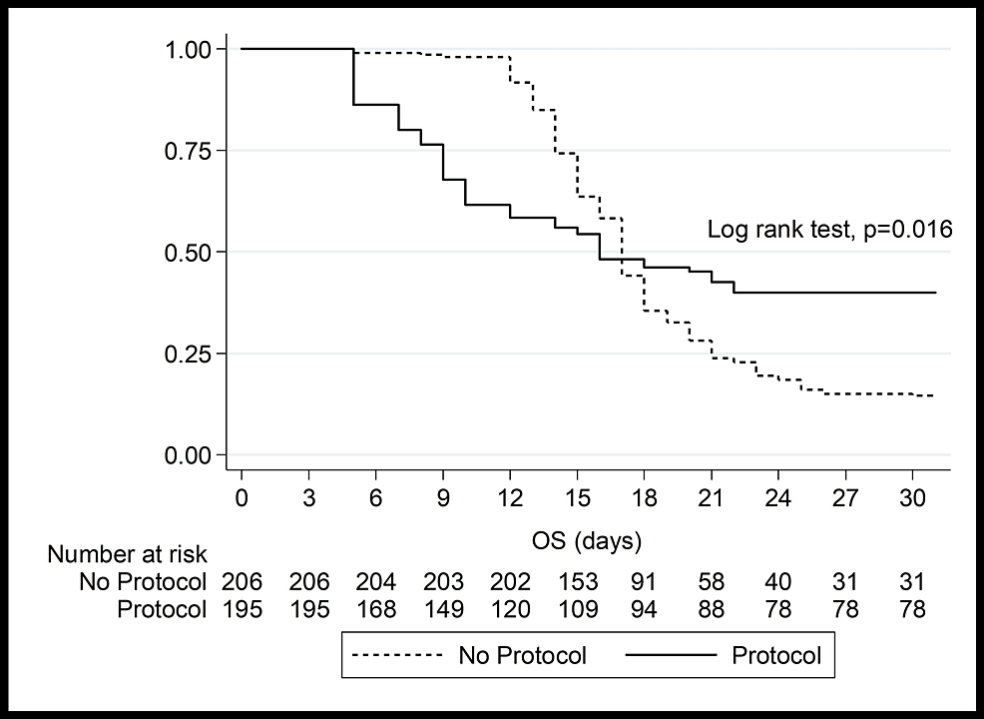
Kaplan-Meier survival estimates: 30-day mortality between the two groups Values are presented as median. p-values were analyzed using the Kaplan-Meier survival estimates.

Period effect analysis findings are displayed in Table 2. Compared with the one-hour bundle group, the usual care group had mean times to initial fluid resuscitation, vasopressor, culture, empirical antibiotic initiation, and source control of 2.33 ± 0.58 hours, 3.52 ± 0.84 hours, 4.17 ± 0.5 hours, 5.16 ± 0.54 hours, and 10.32 ± 0.66 hours, respectively (Table 2). Hospital and ICU lengths of stay were significantly increased in the usual care group (16.8 ± 3.77 days versus 7.91 ± 1.68 days and 12.07 ± 4.38 days versus 3.88 ± 1.37 days, respectively) (p < 0.001). Compared with the one-hour bundle group, the usual care group had a significantly greater cost of treatment (62,919.37 ± 1,186.29 Thai Baht versus 48,005.35 ± 9,598.83 Thai Baht, respectively) (p < 0.001). Compared with the one-hour bundle group, the usual care group had significantly higher mortality days (6.43 ± 6.63 days versus 14.27 ± 6.89 days, respectively) (p < 0.001). Compared with the usual care group, the one-hour bundle group had significantly higher ventilator- and vasopressor-free days (5.59 ± 1.9 days versus 1.67 ± 0.82 days and 6.25 ± 1.81 days versus 3 days, respectively) (p < 0.001). After the one-hour bundle protocol was applied to practice, the median survival time was significantly increased in the one-hour bundle group (95% confidence interval (CI): 12.32-19.68) (p = 0.016) (Figure 1). After the one-hour bundle protocol was applied to practice, initial fluid resuscitation, vasopressor, blood culture and sensitivity, and empirical antibiotic initiation were implemented within one hour of the diagnosis of sepsis and septic shock based on the Sepsis-3 guideline, and source control was performed within 6-12 hours (Table 3). Each result classified by time to perform in the usual care group was demonstrated (Table 3).

**Table 3:** Difference in secondary outcomes between the two patient groups Values are presented as median. p-values were analyzed using the Mann-Whitney test and chi-square test.

	One-hour bundle (n = 195)	Usual care (n = 206)	p-value
Initial fluid resuscitation (hours)			
1	195 (100%)	5 (2.4%)	<0.001*
2	0 (0%)	134 (65%)	
3	0 (0%)	60 (29.1%)	
4	0 (0%)	7 (3.4%)	
Vasopressor (hours)			
1	195 (100%)	2 (1%)	<0.001*
2	0 (0%)	3 (1.5%)	
3	0 (0%)	114 (55.3%)	
4	0 (0%)	68 (33%)	
5	0 (0%)	10 (4.9%)	
6	0 (0%)	9 (4.4%)	
Culture (hours)			
1	195 (100%)	0 (0%)	<0.001*
2	0 (0%)	2 (1%)	
3	0 (0%)	5 (2.4%)	
4	0 (0%)	155 (75.2%)	
5	0 (0%)	44 (21.4%)	
Initiated empirical antibiotics (hours)			
1	195 (100%)	0 (0%)	<0.001*
2	0 (0%)	1 (0.5%)	
3	0 (0%)	2 (1%)	
4	0 (0%)	4 (1.9%)	
5	0 (0%)	155 (75.2%)	
6	0 (0%)	44 (21.4%)	
Source control (hours)			
6	27 (13.8%)	0 (0%)	<0.001*
1	18 (9.2%)	0 (0%)	
3	58 (29.7%)	0 (0%)	
4	34 (17.4%)	0 (0%)	
5	58 (29.7%)	0 (0%)	
8	0 (0%)	4 (1.9%)	
9	0 (0%)	2 (1%)	
10	0 (0%)	132 (64.1%)	
11	0 (0%)	60 (29.1%)	
12	0 (0%)	8 (3.9%)	

Factors associated with mortality included delayed initial fluid resuscitation (>2 hours), vasopressor use (>2 hours), culture sensitivity (>4 hours), and empirical antibiotic initiation (>5 hours) (p = 0.017, 0.028, 0.008, and 0.008, respectively) (Table 4).

**Table 4:** Univariate and multivariate for Cox regression: factors associated with mortality outcomes Values are presented as median. p-values were analyzed using the Mann-Whitney test, chi-square test, and Cox regression model. HR: hazard ratio, CI: confidence interval

	Univariate		Multivariate	
	HR (95% CI)	p-value	Adjusted HR (95% CI)	p-value
Group				
Usual care	Reference	1	Reference	1
One-hour bundle	0.76 (0.6-0.96)	0.02*	0.44 (0.03-7.93)	0.581
Initial fluid resuscitation (hours)				
1	Reference	1	Reference	1
2	1.37 (1.06-1.77)	0.017*	0.48 (0.03-7.97)	0.611
3	1.15 (0.82-1.61)	0.408	0.55 (0.03-9.55)	0.683
4	1.68 (0.78-3.61)	0.183	0.58 (0.03-10.53)	0.712
Vasopressor (hours)				
1	Reference	1	Reference	1
2	0.64 (0.16-2.6)	0.536	0.04 (0-0.7)	0.028*
3	1.29 (0.98-1.69)	0.069	0.83 (0.02-43.28)	0.928
4	1.42 (1.04-1.94)	0.029*	0.94 (0.02-49.93)	0.976
5	0.97 (0.45-2.08)	0.933	0.66 (0.01-37.15)	0.839
6	1.36 (0.66-2.79)	0.402	0.86 (0.02-48.4)	0.943
Culture (hours)				
1	Reference	1	Reference	1
2	2.35 (0.58-9.55)	0.232		
3	1.22 (0.45-3.3)	0.698	2.32 (0.07-82.06)	0.645
4	1.41 (1.1-1.81)	0.008*	7.56 (0.93-61.59)	0.059
5	1.06 (0.72-1.56)	0.756	1.47 (0.78-2.78)	0.234
Initiated empirical antibiotics (hours)				
1	Reference	1	Reference	1
2	1.97 (0.27-14.15)	0.5	-	-
3	1.4 (0.35-5.67)	0.637	-	-
4	1.36 (0.43-4.29)	0.596	-	-
5	1.41 (1.09-1.81)	0.008*	0.38 (0-50.11)	0.698
6	1.06 (0.72-1.56)	0.758	1.4 (0.08-25.14)	0.818

## Discussion

Surgical sepsis and septic shock are a leading cause of morbidity and mortality. Postoperative surgical sepsis and septic shock are highly prevalent and lethal syndromes. Nowadays, the Surviving Sepsis Campaign has been developed one-hour bundle care, but no study explores the clinical outcomes. In this study, the effectiveness of one-hour bundle care for sepsis and septic shock in surgical patients in the ICU was evaluated and compared with that of usual care in this patient group at Phramongkutklao Hospital. The primary outcome of the study was that a decrease in mortality was associated with the one-hour bundle care protocol. This was accomplished with a reduction in the cost of treatment compared with that of usual care for sepsis and septic shock. Moreover, our results are similar to those of a previous study [20], which showed that the one-hour bundle was a cost-effective alternative to the usual care of sepsis and septic shock in surgical patients. After the one-hour bundle protocol was implemented as a clinical practice guideline in this study, the median survival time significantly increased in the one-hour bundle group. Moreover, ICU length of stay significantly decreased from 11 days to four days, and ventilator- and vasopressor-free days significantly increased from two days to six days and from three days to seven days, respectively. Factors associated with mortality included delayed initial fluid resuscitation (>2 hours), vasopressor use (>2 hours), culture (>4 hours), and empirical antibiotic initiation (>5 hours). Another recent study [21] demonstrated even better results, as the one-hour bundle both improved mortality rates and reduced costs of treatment. Early attempts at fluid resuscitation initially emphasized the provision of supraphysiological levels of tissue oxygen delivery substrate, a concept defined as supranormal oxygen delivery. However, previous studies [22,23] based on the interpretation of data from pulmonary artery catheters and the treatment arm that conducts the liberal use of inotropic support failed to show clinical benefit.

Performing surgery at the source of infection control addresses the root cause of sepsis, septic shock, and sepsis progression to septic shock by decreasing or eliminating the site of infection. Although all of these steps must be completed, surgical patients with sepsis or septic shock patients must first be resuscitated to restore tissue perfusion. Investigations into the origin of the infection can be performed simultaneously at the bedside if possible (e.g., portable ultrasound). Intervention for source control should be performed with the least physiological insult possible, such as that with a percutaneous catheter or intervention for drainage of abscesses, until the patient is hemodynamically stable and able to withstand a more definitive surgical procedure, if necessary. Some specific infection origins, especially necrotizing soft tissue infections such as necrotizing fasciitis and necrotic ischemic bowels, require emergency surgery, necessitating rapid resuscitation and surgical correction without delay once the patient is hemodynamically stable and able to tolerate the induction of general anesthesia [24]. A surgical sepsis and septic shock screening program in conjunction with the one-hour bundle for the delivery of evidence-based clinical practice guidelines and to attempt early source control can improve patient outcomes and decrease hospital mortality.

Limitations

This study had several limitations. First, this was a prospective cohort study, and patients were not randomized into groups, although this study better reflects clinical practice [25]. Second, the data on post-surgical ICU care were not followed after ICU and hospital discharge and were therefore not included in the analysis. Third, the use of glycemic control with insulin therapy was not collected. Fourth, adherence to the one-hour bundle protocol, although significantly higher after SSC clinical practice implementation, was relatively low. The implementation of other evidence-based medicine, such as academic conferences, or morbidity and mortality audits and feedback might have further improved adherence [26]. Nonetheless, better adherence to SSC guidelines may reduce associated costs and mortality owing to surgical sepsis and septic shock. Fifth, to maintain adherence to the one-hour bundle protocol in long-term care, additional impulsive clinical practice guideline efforts might be encouraged, increasing the overall cost. Sixth, this study was conducted at a single center. Finally, subgroup analysis seemed to suggest some differences in the outcomes of the cost-effectiveness profile of the one-hour bundle care protocol for age, sex, and severity. Older, male, and more severely affected patients had a worse cost-effectiveness profile than their counterparts. The heterogeneity of the population in the Thai surgical ICU reflects on the mortality of patients in multiple ways.

## Conclusions

In conclusion, the findings of the present study suggest that the one-hour bundle care protocol is an effective option for treating surgical patients with sepsis and septic shock in the ICU. One-hour bundle care resulted in increased median survival time, time to ventilator use, and vasopressor-free days. Consequently, ICU length of stay is decreased. As performance measures are recommended for improving the management of surgical sepsis, ongoing frequency evaluations for the impact of these measures on clinical outcomes and costs of treatment should be carefully conducted.
